# The House of Steinway

**Published:** 1874-11

**Authors:** 


### THE HOUSE OF STEINWAY.

Under this caption we announced last month our intention to commence in this issue of the Journal a series of article descriptive of the pianos which have made this firm celebrated in all civilized lands and among all cultivated people.

It is evident that no history of the pianoforte could lay claim to anything like completeness, which did not assign to the improvements which the family of Steinway have added to this beautiful instrument, a prominent place. It is safe to aver that much of the popularity of the piano in America is due to the honesty of construction and the superior excellence of the products of the manufactory of the Steinways.

It is curious to look back and observe the small beginning of the great industries after they have achieved pronounced success. It is instructive and encouraging to note the gradual but steady and sure growth which usually attends the earnest laborer in any field of enterprise in which he works with industry and enthusiasm. The first piano made by the Steinways was constructed in 1853, in a little shop in Varick Street. It was a good instrument for the period, and a purchaser was speedily found for it. The business of making pianos was then entered upon systematically, and resulted in the production of one hundred excellent instruments during the next two years. Then, by the simple accident of the exhibition of one of their pianos in the New York Crystal Palace, they were made famous in a day. From obscurity they rose at once to fame. The judges awarded to the instrument of these obscure and almost unknown artizans the first prize. From that moment their fortune was assured.

It was not by any influence other than legitimate that this result was reached. There were many pianos on exhibition, and some of them were by celebrated and wealthy makers. But the unpretending instrument bearing the name of Steinway had superior merit, and the honest judges promptly admitted the fact.

But the advantages which were secured by the improvements of these new builders were not simply such as were apparent at the moment. In adding to the melody they had greatly prolonged the lifetime of the instrument. This fact could only be demonstrated after years of practical service. To provide a framework capable of resisting the enormous strain or pull of the many wires when the tension was complete had exhausted the inventive resources of all builders since the first rude pianoforte was made by Cristofali, of Florence, in 1710. What difficulties beset the artisan in this direction may be imagined, when we state that the tensile force, or lifting power, exerted by the smallest and shortest wire, when keyed up to concert pitch, is two hundred and sixty pounds; and that the aggregate strain upon all the strings of a grand piano, if used for the purpose of elevating a burden, would suffice to raise a weight of twenty tons. This pull, equal to the power of twenty strong horses, must be kept up constantly year after year. If a single wire of the many becomes slackened and listless, if a single pound of force is withdrawn, the delicate tones are all ajar, "like sweet bells jangled, out of tune," and discord reigns until the old tension is restored. This tendency to slacken was an inherent fault in the pianos made prior to the advent of the Steinways. For a piano to be "out of tune" was a thing of course. The annual cost of tuning an instrument was at one period much greater than the interest on the money invested in the purchase. The trouble was that the structure within the box, or outer shell, was too slight to permanently sustain the enormous strain. Something must give way, and it was safer to let the strings slip and slacken than to fasten them so securely as to destroy the structure. All these difficulties increased as the scale of pianos was enlarged. To secure the best musical effects it was found necessary to extend the compass until it reached seven and one-third octaves. The old wooden bottoms, made of timbers five inches thick, were inadequate to sustain the combined strain of this mass of wires. Iron supports had previously been used to a limited extent, but the employment of this metal had been attended with a thin and nasal quality of tone. To avail themselves of the strength of iron, and at the same time to retain the resonance of wood, was the work of the Steinways. With consummate art they constructed a full iron frame with a strong bar in front, and with such an adjustment of braces as to completely antagonise the enormous strain and secure the needed resistance, while greatly lessening the bulk; retaining, by the interposition of wooden bridges, the volume and resonance of tone which the use of iron had in other hands destroyed. This employment of iron in conjunction with wood was the first great step towards the wonderfully perfect instruments, for the production of which the House of Steinway has attained its world-wide celebrity.

In future numbers of the Journal we will notice other improvements in the construction of pianos, and will endeavor to give our readers some idea of the vastness of this branch of industry.

